# Verbal Fluency Is Affected by Altered Brain Lateralization in Adults Who Were Born Very Preterm

**DOI:** 10.1523/ENEURO.0274-18.2018

**Published:** 2019-04-15

**Authors:** Chieh-En Jane Tseng, Sean Froudist-Walsh, Jasmin Kroll, Vyacheslav Karolis, Philip J. Brittain, Nadia Palamin, Hayley Clifton, Serena J. Counsell, Steven C. R. Williams, Robin M. Murray, Chiara Nosarti

**Affiliations:** 1 Department of Psychosis Studies, Institute of Psychiatry, Psychology and Neuroscience, King’s College London, London, United Kingdom SE5 8AF; 2 Center for Neural Science, New York University, New York, NY 10003; 3 Department of Developmental and Social Psychology (DPSS), University of Padova, 35131, Padova, Italy; 4 Centre for the Developing Brain, Division of Imaging Sciences & Biomedical Engineering, King’s College London, London, United Kingdom SE1 7EH; 5 Centre for Neuroimaging Sciences, Institute of Psychiatry, Psychology and Neuroscience, King’s College London, London, United Kingdom SE5 8AF

**Keywords:** fMRI, lateralization, verbal fluency, very preterm

## Abstract

Language difficulties have been reported in children and adolescents who were born very preterm (<32 weeks’ gestation) and associated with an atypical lateralization of language processing, i.e., increased right-hemispheric engagement. This study used functional magnetic resonance imaging (fMRI) and spherical deconvolution tractography to study the hemodynamic responses associated with verbal fluency processing (easy and hard letter trials) and verbal fluency-related white matter fiber tracts in 64 very preterm born adults and 36 adult controls (mean age: 30 years). Tractography of the arcuate fasciculus (AF) and frontal aslant tract (FAT) was performed. Tracts were quantified in terms of mean volume, hindrance modulated orientational anisotropy, and lateralization, assessed using a laterality index (LI) to indicate hemispheric dominance. During verbal fluency fMRI, very preterm participants displayed decreased hemodynamic response suppression in both the Easy > Rest and Hard > Rest conditions, compared to controls, in superior temporal gyrus (STG), insula, thalamus, and sensorimotor cortex, particularly in the right hemisphere. At the whole-group level, decreased hemodynamic response suppression in the right sensorimotor cortex was associated with worse on-line performance on the hard letter trials. Increased left-laterality in the AF was present alongside increased right hemispheric hemodynamic response suppression in controls. When only right-handed participants were considered, decreased hemodynamic response suppression in the right STG during hard letter trials was related to weaker left and right FAT white matter integrity in the preterm group only. These results show that verbal fluency is affected by altered functional lateralization in adults who were born very preterm.

## Significance Statement

This is the first study to use both functional and structural magnetic resonance imaging (MRI) to assess the neuroanatomy of verbal fluency in very preterm born adults. Less suppression of brain activation was observed in very preterm adults compared to controls in several brain regions during completion of both easy and hard verbal fluency trials. Furthermore, across all subjects, decreased brain activity suppression in the right sensorimotor cortex was associated with worse on-line performance on the hard letter trials. Increased left-laterality in the arcuate fasciculus (AF), a language-related white matter tract, was present alongside increased right hemispheric brain activity suppression in controls. These findings suggest that alterations in the typical development of left-lateralization in very preterm individuals are still present in adulthood.

## Introduction

During the third trimester of pregnancy, the fetal brain more than doubles in size and the volume of cortical gray matter increases approximately four-fold (Hüppi et al., 1998). At the same time, thalamocortical axons are reaching the cortical plate and callosal white matter connections are spreading across the subplate zone (Kostovíc and Jovanov-Milosevíc, 2006). These processes establish the neural foundation for the development of cognitive and motor functions. Very preterm birth (<32 weeks’ gestation) can thus lead to a complex pattern of exogenous and endogenous insults (Volpe, 2009), which result in alterations to structural and functional brain development (Smyser et al., 2010; Ball et al., 2015).

In terms of cognitive outcomes, very preterm born individuals have shown poorer verbal fluency performance than controls (Aarnoudse-Moens et al., 2009; Nam et al., 2015). Verbal fluency involves strategic search and retrieval processes from lexicon and semantic memory (Sauzéon et al., 2004), which tests both verbal ability and executive control. Impairments in such domains are believed to affect academic achievement and may lead to poorer occupational prospects (Kroll et al., 2017). While receptive language abilities have been shown to improve with age in very preterm children, deficits in expressive language functions seem to persist into adolescence (Luu et al., 2011). Using functional magnetic resonance imaging (fMRI), it was previously demonstrated that while completing a verbal fluency task with different cognitive loads, very preterm young adults showed differences in hemodynamic response compared to controls predominantly in frontal, parietal, temporal, and subcortical regions (Nosarti et al., 2009; Kalpakidou et al., 2014).

Several studies described structural and functional brain asymmetries of language-related regions during typical development (Sowell et al., 2002; Dehaene-Lambertz et al., 2006a,b; Friederici et al., 2011; Kasprian et al., 2011). A deeper right superior temporal sulcus and larger left temporal lobe was observed as early as 23 weeks’ gestation (Kasprian et al., 2011). This asymmetry continues to develop postnatally, with perisylvian sulcal asymmetries being more prominent in adults than in children (Sowell et al., 2002). fMRI studies demonstrated dominant left-hemispheric responses during processing of language-related auditory stimuli in newborn infants (Dehaene-Lambertz et al., 2006a,b). However, a lack of lateralization in language related regions was observed in very preterm infants at term equivalent age compared to term control infants (Kwon et al., 2015).

Increased left-lateralization in language homologs may reflect typical maturational processes from childhood to adulthood (Friederici et al., 2011). This process may be altered in very preterm individuals, as increased right-hemispheric engagement was found in very preterm adolescents during a verbal task (Gozzo et al., 2009; Myers et al., 2010), suggesting the use of alternate neural pathways for language processing. However, this alternative neural pathway could be suboptimal, given the finding that stronger right-lateralization in very preterm adolescents was associated with poorer language performance (Scheinost et al., 2015).

Measures of language have also been related to microstructural integrity of white matter connections in preterm samples, and similarly to fMRI studies, show a bilateral language network (Mullen et al., 2011; Feldman et al., 2012). The arcuate fasciculus (AF) and the frontal aslant tract (FAT) are two white matter tracts that are involved in the verbal component of verbal fluency. The AF connects the superior temporal gyrus (STG) to the inferior frontal gyrus (IFG) and has long been recognized for its involvement in language. The FAT is a recently identified pathway that connects the supplementary motor area to the IFG (Catani et al., 2012). It has been shown to be involved in speech fluency in adults who stutter (Kronfeld-Duenias et al., 2016) and individuals with primary progressive aphasia (Catani et al., 2013).

This study tested the following hypotheses: (1) during completion of a verbal fluency task, very preterm adults would display a greater recruitment of homologous language-related regions in the right hemisphere in comparison to controls; (2) very preterm adults would exhibit smaller volume and hindrance modulated orientational anisotropy (HMOA; a tract-specific characterization of white matter microstructure) and decreased left-lateralization in the structural indices of the AF and FAT tracts compared to controls; and (3) increased right hemispheric hemodynamic response in very preterm adults would be associated with worse verbal fluency performance and stronger right-lateralization in white matter structural indices. We further explored possible between-group differences in the associations between fMRI data and task performance and white matter tract measurements to evaluate whether (1) they would show the same pattern in very preterm born adults and controls, or (2) they would show different associations in the two participant groups.

## Materials and Methods

Participants were part of a larger study that followed up a cohort of individuals born at <33 weeks of gestation who were admitted to the neonatal unit of University College Hospital, London, between 1979 and 1985. Term born control participants were recruited from the community and were matched in age to very preterm adults. Inclusion criteria were full-term birth (38–42 weeks), birth weight >2500 g, and age between 28 and 35 years. Exclusion criteria for the control group included birth complications (e.g., low birth weight defined as <2500 g, endotracheal mechanical ventilation), prolonged gestation (>42 weeks), severe hearing and motor impairments, and mental retardation indicated by intelligence quotient (IQ) < 70. All study participants were native English speakers. Among these participants, 64 very preterm participants and 36 controls of either sex were assigned at random to complete a verbal fluency fMRI task.

IQ was assessed using the Wechsler Abbreviated Scale of Intelligence (WASI; Wechsler, 1999), which consists of four subtests that estimate verbal IQ, performance and full-scale IQ. Participants’ handedness was assessed using the Modified Annett Questionnaire (Annett, 1967). The threshold used was which hand participants reported using in more than four out of six questions. Participants gave full informed consent and the study was approved by the appropriate local ethics committees, and in compliance with national legislation and the code of ethical principles for Medical Research Involving Human Subjects of the World Medical Association (Declaration of Helsinki).

Neonatal and socio-demographic information for all participants is shown in Table 1. Very preterm adults were slightly older and had lower verbal IQ scores than controls. Hence age was accounted for in all further analyses. Verbal IQ was not controlled for as it was assumed to share variance with the effect of interest. Performance IQ was not significantly different between the groups. There were no significant between-group differences in sex, socioeconomic status (Her Majesty's Stationary Office, 1991) , or handedness.

**Table 1.** Participants’ neonatal and socio-demographic variables

	Very preterm (*n* = 64)	Control (*n* = 36)	Test statistic	*p* value
Age (mean ± SD)	31.53 ± 2.44	30.47 ± 6.36	*U* = 806.0	**0.013**
Sex (M/F)	36/28	21/15	χ^2^ = 0.041	1.000
IQ				
Verbal IQ	97 ± 18.37	107.73 ± 16.33	*U* = 1159.5	**0.017**
Performance IQ	104.95 ± 14.90	109.72 ± 15.59	*U* = 1017.5	0.112
Gestational age	29.48 ± 1.98	--	--	--
Birthweight	1311.12 ± 376.41	--	--	--
Neonatal ultrasound (brain injury/normal)^a^	28/36	--	--	--
Handedness (L/R/A)^b^^	11/52/1	1/28/0	Fisher’s exact = 3.838	0.12
Socioeconomic status^*a^				
I-II (professional and Intermediate)	27	15	Fisher’s exact = 5.195	0.241
III (skilled manual and non-manual)	26	15		
IV-V (semi-skilled and unskilled manual)	2	0		
Students	1	4		
Unemployed	7	2		

*p* values that remained significant after FDR correction are indicated in bold.

* Her Majesty’s Stationary Office (1991), missing information for one participant.

a Neonatal brain injury includes uncomplicated periventricular hemorrhage without ventricular dilation and periventricular hemorrhage with ventricular dilation (Stewart et al., 1983).

b Fisher’s exact test.

^ Missing information for seven control participants.

### Phonemic verbal fluency task

**Figure 1.**
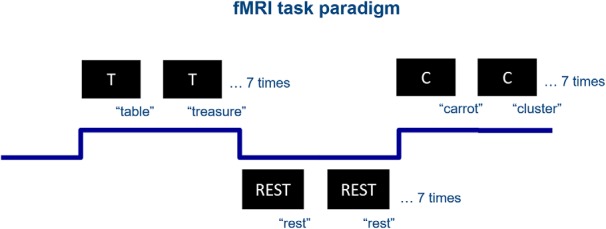
Verbal fluency fMRI task paradigm.

The fMRI task used in this study was a well-validated phonemic verbal fluency paradigm (Fu et al., 2002). Participants were required to overtly generate a word starting with the letter presented on a computer screen projected into the MRI scanner, but to not use proper names, grammatical variation of the previous word, or to repeat previous responses. If participants were unable to generate a response, they were asked to say “pass.” Each letter was presented seven times within each block for a total of ten blocks, each block lasted 28 s (Fig. 1). The “easy” letters were: T, C, B, P, S; and the “hard” letters were: I, N, F, E, G. The categorization of easy and hard letters was based on the mean number of erroneous responses generated for each letter in a previous study (Fu et al., 2002). A 2 s “silent” period was set to allow for the participant to respond, coupled with a 2 s image volume acquisition period. During the “rest” blocks, participants were presented with the word rest and required to say rest out loud. The rest blocks were of the same duration as the task blocks. Verbal responses were recorded through an MRI-compatible microphone on Cool Edit 2000 (Syntrillium Software Corporation). Verbal fluency performance was assessed by the accuracy rate of participants’ response (i.e., correctly producing a word starting with the indicated letter; not using proper names, grammatical variation of the previous word, or saying pass). Participants were familiarized with the task before the fMRI experiment in an offline training session in which they were asked to make responses to example trials using a different set of letters.

### Image acquisition

Data were collected using a GE 3 tesla Signa MR scanner (GE Healthcare). A gradient-echo EPI sequence (TR/TE = 2000/30 ms) was used to collect data from 36 non-contiguous slices of 3.5 mm thickness separated by a distance of 0.5 mm, and with in-plane voxel resolution of 3.75 × 3.75 mm^2^. These were co-registered with T1-weighted anatomic image (TR/TE/TI: 7.1/2.8/450 ms, matrix: 256 × 256), allowing for 196 slices with no gap and an isotropic resolution of 1.1 × 1.1 × 1.1 mm^3^.

Diffusion-weighted images were acquired using a multi-slice spin echo EPI sequence (TE = 104.5 ms), obtaining 60 contiguous near-axial slice locations with isotropic (2.4 × 2.4 × 2.4 mm^3^) voxels. The b value was 1300 s/mm^2^, with 32 diffusion-weighted directions and four non-diffusion-weighted volumes. Peripheral cardiac gating was applied with an effective TR of 20/30 RR interval.

### fMRI analysis

Statistical analysis of fMRI data was performed using FEAT from FSL (FMRIB Software Library; http://www.fmrib.ox.ac.uk/fsl). The three initial volumes were removed to minimize the effects of magnetic saturation. Pre-processing steps included motion correction (FSL’s FLIRT), time-slice correction, spatial smoothing (Gaussian, FWHM 5 mm), and temporal high-pass filtering (sigma = 50 s). There were no statistically significant differences between very preterm and control participants in head motion during the fMRI task (*U* = 1111, *p* = 0.59). Denoising was performed using FSL’s independent component analysis ICA-based Xnoiseifier (FIX; Griffanti et al., 2014; Salimi-Khorshidi et al., 2014). The components of 20 participants (10 very preterms and 10 controls) were identified manually as noise or non-noise components according to established guidelines (Kelly et al., 2010). This information was used to train a classifier that can automatically classify the ICA components of each participant into noise or non-noise components. The time courses of the noise components were regressed out of the data. Regressors for each condition in the general linear model were convolved with a gamma hemodynamic response function. Only correct responses were used for the analyses. Individual participant data were then entered into a higher-level analysis using a mixed effects design (FLAME) whole-brain analysis and age was added as a covariate.

Three contrasts were studied: Easy > Rest, Hard > Rest, and Hard > Easy. Cluster-based thresholding was used to find significant clusters. *Z*-statistic maps were thresholded at *z* = 2.3. Voxels that pass the threshold formed clusters, and the spatial extent of each cluster was calculated. Then, random field theory was used to find the *p* value of obtaining a cluster of a spatial extent given the chosen *z*-threshold and the spatial smoothness of the noise in the data under the null hypothesis. These *p* values were corrected for family wise error across voxels and a threshold of *p* < 0.05 was used to obtain significant clusters. From the resulting cluster maps, we identified clusters of hemodynamic response that significantly differed between groups, after controlling for participants’ age. No significant results were found when comparing very preterm adults with brain injury, very preterm adults with normal ultrasound classification (subgrouped according to neonatal ultrasound [Stewart et al., 1983]) and controls; therefore, we focused on comparisons between all very preterm individuals and controls. In addition to exploring between-group differences in hemodynamic response, we also investigated whether hemodynamic response in brain areas displaying significant between-group differences was associated with on-line task performance and white matter tract characteristics. This was done by obtaining cluster masks of regions displaying significant between-group differences in hemodynamic response and extracting the parameter estimates of each individual.

### Normalization

Each individual’s functional data were registered to their structural scan using FSL’s FLIRT (Jenkinson and Smith, 2001; Jenkinson et al., 2002) and boundary-based registration (BBR) cost function (Greve and Fischl, 2009). This technique extracts the surfaces from the T1-weighted image, and then aligns the fMRI data to the T1-weighted data by maximizing the intensity gradient across tissue boundaries. This method has been shown to be more accurate and robust to signal inhomogeneities than traditional intrasubject registration algorithms. To map each individual’s data into a common space, we used FSL-FNIRT (Andersson et al., 2010) to normalize each individual’s structural data to a study-specific template, which is an average of 78 brain images from term-born and very preterm individuals as used in Froudist-Walsh et al. (2015; available on request).

### Tractography analysis

Preprocessing of diffusion MRI data followed the pipeline developed by Froudist-Walsh et al. (2015). Brain extraction was performed on the diffusion-weighted and b0 images using FSL’s BET. Motion and eddy-current corrections were done on the brain-extracted data using ExploreDTI (Leemans et al., 2009). This motion correction step realigns the images and reorients the B-matrix so that the correct orientational information is preserved (Leemans and Jones, 2009). There were no statistically significant differences between very preterm and control participants in head motion in the diffusion data (*U* = 1044, *p* = 0.84). A constrained spherical deconvolution approach was chosen to differentiate multiple directions within one voxel (Tournier et al., 2004). We chose this approach as tractography using constrained spherical deconvolution outperforms tractography using other reconstruction methods when using data acquired with clinical b values (Wilkins et al., 2015). Constrained spherical deconvolution was performed using a damped version of the Richardson–Lucy algorithm (Dell'acqua et al., 2010). Parameters were chosen based on recommendations from the StarTrack manual (https://www.mr-startrack.com/) and by visual inspection of the reconstruction to find the best possible balance between resolving multiple fiber orientations and minimizing false-positive fiber orientation distributions (FODs). The parameters used were: regularization threshold η = 0.02, fiber response function α = 2, algorithm iterations = 300, and regularization parameter v = 20; which is what was used in previous studies in the same cohort (Froudist-Walsh et al., 2015; Karolis et al., 2016; Tseng et al., 2017).

Visual inspection was performed in regions with known crossing fibers (e.g., between the corpus callosum, superior longitudinal fasciculus, and corticospinal tract) and without (e.g., middle of the corpus callosum).

Fiber orientation estimates were taken from the orientation of the peaks of the FOD profile. We used an absolute (equal to four times the amplitude of a spherical FOD obtained from a gray matter voxel) and a relative threshold (equal to 7% of the amplitude of the maximum amplitude of the FOD at that voxel) at each voxel to remove the general noise floor and surviving noise local maxima, respectively. Each FOD that survived the threshold were used as seeds to perform whole-brain tractography. Fiber orientation streamlines were propagated using Euler integration with a step size of 1 mm. Propagation stopped if the angle between two successive steps exceeded 60°. As the AF is a curved bundle, a more lenient angular threshold was used to ensure the AF could be reconstructed in all participants. This threshold is also close to that used by Phillips et al. (55°) to preclude the generation of fibers with biologically unrealistic curvature (i.e., “looping” fibers; Phillips et al., 2012). Tractography reconstruction was performed using StarTrack (Dell'Acqua et al., 2013). The final reconstructed whole-brain tractography was visually assessed for all participants.

**Figure 2.**
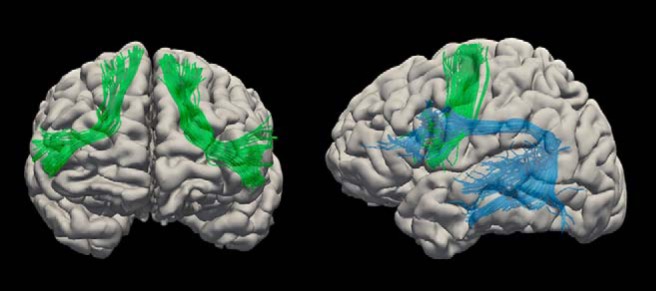
The arcuate fasciculus (blue) and frontal aslant tract (green).

White matter dissection of the AF and FAT were performed in native diffusion space in TrackVis (http://www.trackvis.org) using a two-region method (Catani and Thiebaut de Schotten, 2008; Catani et al., 2012). In this study, we only considered the long segment of the AF, which is the only bundle that arches around the Sylvian fissure to connect posterior temporal regions to the IFG. The AF was identified using regions of interest (ROI) of the IFG and posterior STG and middle temporal gyrus (MTG). Tracts that passed through these ROIs, but originated from the anterior temporal regions, were excluded in order not to include the middle longitudinal temporal parietal tracts. The FAT was identified using ROIs of the IFG (defined as BA45 and 44) and posterior superior frontal gyrus. All ROIs were hand drawn for each participant and all tracts were dissected in both hemispheres. Artefactual/non-anatomic fibers were removed using manually drawn region-of-avoidances based on the literature of brain anatomy and shape of the tract (Catani et al., 2012; Dick and Tremblay, 2012). An example of the dissected tracts is shown in Figure 2. White matter tracts were evaluated by HMOA and volume. White matter tract volumes were adjusted for intracranial volume by dividing tract volume by intracranial volume. Age was controlled for in all white matter variables using robust regression and a logistic weight function in MATLAB (MATLAB and Statistics Toolbox Release R2014b, The MathWorks, Inc.), the residuals were then used for further statistical analysis described below.

### Lateralization

Laterality index (LI) of the white matter tract measures (HMOA, volume, FA, RD) were obtained by LI = (Q_left_-Q_right_)/(Q_left_+Q_right_) (Seghier, 2008).

### Statistical analysis of demographic, behavioral, and IQ data and integration of imaging-derived measures

Statistical analyses were done in SPSS 21 (IBM SPSS Statistics for Macintosh, version 21.0). The distributions of the imaging (fMRI and white matter tract measurements: left and right AF HMOA and volume, left and right FAT HMOA and volume, and LI of AF and FAT HMOA and volume), demographic (age, gestational age, birth weight), behavioral (verbal fluency task performance, head motion), and verbal and performance IQ data were tested for normality using a Shapiro–Wilk test. Not all variables were normally distributed; therefore, group comparisons were performed using Mann–Whitney *U* tests and correlation tests were performed using Spearman’s correlation. To explore possible between-group differences in the associations between fMRI data and task performance and white matter tract measurements, all analyses were performed first across the whole sample, then within each group (control and very preterm). After identifying significant within-group associations, interaction terms were included in a univariate linear regression analysis to test for between groups differences in such associations. Multiple comparison correction was performed using false discovery rate (FDR; Benjamini and Hochberg, 1995). to investigate whether verbal fluency performance was driven by between-group differences in verbal IQ, additional analyses were performed to evaluate the relationship between verbal fluency and verbal IQ.

## Results

### Verbal fluency performance

Very preterm adults performed significantly worse than controls on the hard letter trials (*U* = 1449.5, *p* < 0.001) but not the easy letter trials of the on-line verbal fluency task (*U* = 1647.0, *p* = 0.032, non-significant after FDR correction). There were no statistically significant group differences in correct response times for both easy and hard letters (Table 2).

**Table 2.** Participants’ on-line verbal fluency performance

	Very preterm	Control	Test statistic	*p* value
Task performance	Accuracy (mean ± SD)			
Easy letters	0.83 ± 0.15	0.89 ± 0.10	*U* = 1449.5	0.032
Hard letters	0.70 ± 0.17	0.83 ± 0.13	*U* = 1647.0	**<0.001**
Correct response time	Milliseconds (mean ± SD)			
Easy letters	660.04 ± 159.11	640.83 ± 197.53	*U* = 875.0	0.759
Hard letters	636.73 ± 156.34	610.17 ± 180.78	*U* = 905.0	0.561

*p* values that remained significant after FDR correction are indicated in bold.

### fMRI analysis

Group main effect on the Easy > Rest condition showed positive hemodynamic responses in bilateral paracingulate gyrus, superior, middle, inferior frontal gyrus, anterior insula, caudate, intracalcarine cortex, cerebellum, left precentral gyrus, superior parietal lobule, supramarginal gyrus, putamen, thalamus, middle and inferior temporal gyrus, and lateral occipital cortex (LOC) in both very preterm adults and controls. Very preterm adults also showed positive hemodynamic responses in right precentral gyrus, putamen, and thalamus. The Hard > Rest condition showed similar patterns of positive hemodynamic responses with additional involvement of bilateral STG and right supramarginal gyrus and inferior temporal gyrus. When looking at group main effect on the Hard > Easy condition, the control group showed positive hemodynamic responses in the left LOC.

**Figure 3.**
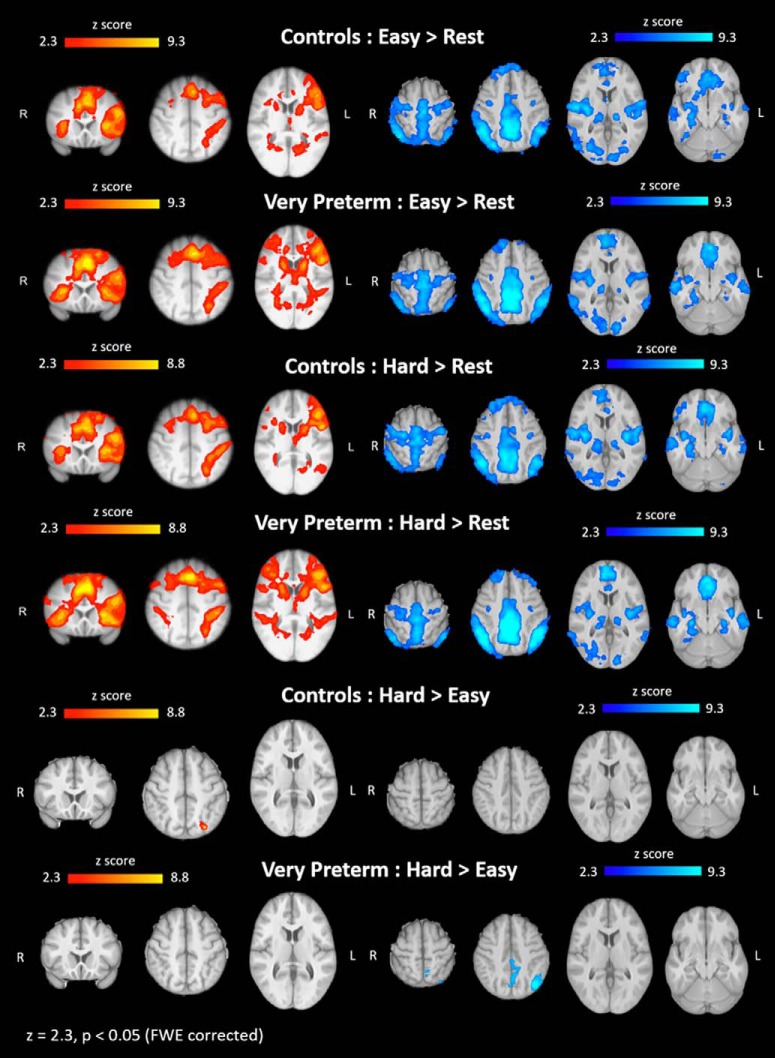
Hemodynamic responses in very preterm adults and controls during easy and hard letter trials. Positive hemodynamic response clusters are shown in red-yellow, negative hemodynamic response clusters are shown in blue-light blue. FWE = family wise error.

The very preterm group did not show any regions displaying positive hemodynamic response (Table 3; Fig. 3).

**Table 3.** Hemodynamic responses in very preterm adults and controls during easy and hard letter trials

Condition		Region	Peak MNI coordinate [*x*,*y*,*z*] (mm)^a^	Cluster size (voxels)^*^
Control Easy > Rest	Positive hemodynamic response	Bilateral paracingulate gyrus, SFG, MFG, IFG, anterior insula, caudate, intracalcarine cortex, cerebellum; left precentral gyrus, putamen, thalamus	[–50, 10, 30] [36, 10, 32] [–6, 10, 60] [–4, 16, 46] [8, 30, 34] [–42, 2, 26]	114,161
		Left SPL, SMg, LOC	[–48, –38, 40]	8772
		Left STG, ITG	[–48, –50, –10]	3073
	Negative hemodynamic response	Bilateral precuneus/PCC, IPL, insula, LOC, sensorimotor cortex, ACC, SFG, thalamus, occipital fusiform gyrus, lingual gyrus, hippocampus, parahippocampus, amygdala; right frontal pole, MTG	[–1, –49, 27] [7, –53, 27] [6, –65, 28] [52, –56, 28] [57, –60,28] [–55, –60, 33]	257,987
		Left cerebellum	[–27, –40, –52]	2701
		Left MTG	[–52, 3, –15]	2690
Very preterm Easy > Rest	Positive hemodynamic response	Bilateral paracingulate gyrus, SFG, MFG, IFG, precentral gyrus, anterior insula, caudate, putamen, thalamus, intracalcarine cortex, cerebellum; left STG, ITG	[–8, 18, 40] [2, 20, 46] [–46, 2, 26] [–52, 2, 22] [–6, 14, 52] [–4, 18, 48]	188,520
		Left SPL, SMg, LOC	[–30, –68, 46]	12146
	Negative hemodynamic response	Right PCC, precuneus, sensorimotor cortex	[4, –50, 30]	79420
		Right LOC, SMg, AG, insula, MTG, putamen, thalamus	[49, –68, 34]	76944
		Left LOC, SMg, AG, insula, MTG	[–54, –62, 34]	46079
		Bilateral ACC, SFG	[–2, 52, 2]	30281
		Left occipital fusiform gyrus, lingual gyrus, parahippocampus, thalamus	[–14, –88, –12]	8467
		Left cerebellum	[–24, –75, –35]	1815
Control Hard > Rest	Positive hemodynamic response	Bilateral paracingulate gyrus, SFG, MFG, IFG, precentral gyrus, anterior insula, caudate, putamen, intracalcarine cortex, cerebellum	[–50, 6, 32] [–50, 14, 28] [–44, 24, 18] [–6, 12, 56] [–2, 16, 46]	125,306
		Left SPL, SMg, LOC	[–46, –40, 38]	13,728
		Left ITG	[–40, –60, –8]	4259
		Right MFG	[40, 40, 36]	2731
	Negative hemodynamic response	Bilateral PCC, precuneus, sensorimotor cortex; right LOC, SMg, AG, insula, MTG, hippocampus, parahippocampus, amygdala, occipital fusiform gyrus, lingual gyrus, putamen, thalamus	[10, –56, 28] [48, –60, 28] [48, –53, 20] [48, –60, 38] [52, –56, 32]	164,196
		Bilateral ACC, SFG; right MFG	[4, 44, 4]	33,368
		Left LOC, SMg, AG,	[–52, –61, 32]	15,964
		Left insula	[–38, –20, 20]	15,701
		Left cerebellum, occipital fusiform gyrus	[–30, –74, –36]	9585
		Left MTG	[–57, 0, –26]	6999
		Bilateral cerebellum	[6, –38, –52]	4063
		Left thalamus	[–15, –26, 3]	2301
		Right frontal pole	[44, 42, –15]	1818
Very preterm Hard > Rest	Positive hemodynamic response	Bilateral paracingulate gyrus, SFG, MFG, IFG, precentral gyrus, anterior insula, caudate, putamen, intracalcarine cortex, STG, ITG, cerebellum; left SPL, SMg, LOC	[–6, 12, 52] [–42, 4, 28] [–8, 22, 40] [–6, 18, 48] [2, 18, 48]	215,947
		Right SMg	[50, –34, 48]	2236
	Negative hemodynamic response	Bilateral PCC, precuneus, sensorimotor cortex; right frontal pole, LOC, SMg, AG, insula, MTG, occipital fusiform gyrus, lingual gyrus, parahippocampus, hippocampus, amygdala, putamen, thalamus	[12, –62, 28] [8, –64, 28] [8, –52, 29] [–10, –50, 39] [–5, –48, 38]	144,407
		Left LOC, SMg, AG, insula, MTG, occipital fusiform gyrus, lingual gyrus, parahippocampus, hippocampus, amygdala, putamen, thalamus	[–49, –59, 38]	53,775
		Bilateral ACC, SFG, MFG	[–5, 52, 18]	36,855
		Bilateral cerebellum	[–9, –46, –46]	2930

a Sub-peaks are only reported for clusters larger than 100,000 voxels.

* All clusters were obtained with *z* = 2.3, *p* < 0.05 (corrected for family wise error across voxels).

SFG = superior frontal gyrus; MFG = middle frontal gyrus; IFG = inferior frontal gyrus; SPL = superior parietal lobule; SMg = supramarginal gyrus; AG = angular gyrus; PCC = posterior cingulate cortex; MTG = middle temporal gyrus; ITG = inferior temporal gyrus, LOC = lateral occipital cortex.

Group main effect on the Easy > Rest condition showed hemodynamic response suppression (i.e., a less negative hemodynamic response) in bilateral precuneus/posterior cingulate cortex (PCC), inferior parietal lobule, occipital fusiform, lingual, superior and middle temporal gyri, insula, lateral occipital, sensorimotor, anterior cingulate cortices, superior frontal gyrus, thalamus, hippocampus, parahippocampus, amygdala, right putamen, and left cerebellum in both very preterm and control participants. Control participants also showed hemodynamic response suppression in the right frontal pole, while very preterm adults showed hemodynamic suppression in the left putamen. Group main effect on the Hard > Rest condition showed hemodynamic response suppression in similar regions as well as the right cerebellum. Very preterm adults had increased suppression in the left middle frontal gyrus. On the Hard > Easy condition, the control group showed no regions of hemodynamic response suppression. The very preterm group showed hemodynamic response suppression in bilateral precuneus, left PCC and LOC (Table 3; Fig. 3).

**Figure 4.**
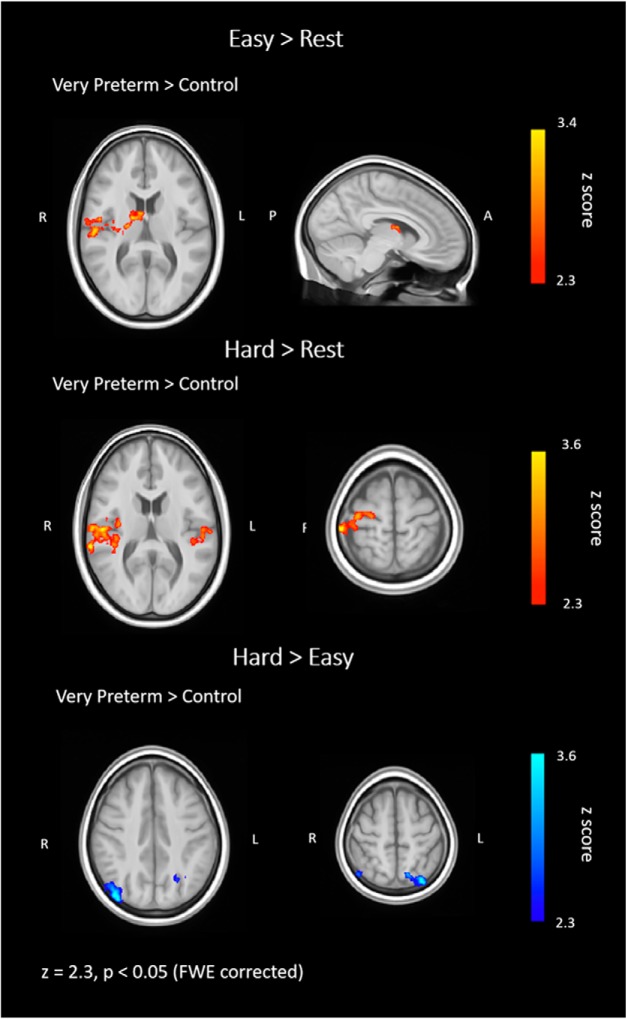
Differences in hemodynamic response between very preterm adults and controls during Easy > Rest, Hard > Rest, and Hard > Easy conditions. Red-yellow indicates relatively increased hemodynamic response in the very preterm group compared to controls, while blue indicates relatively decreased hemodynamic response in the very preterm group compared to controls. FWE = family wise error.

When comparing the hemodynamic responses between groups, very preterm participants showed decreased hemodynamic response suppression in both the Easy > Rest and Hard > Rest conditions compared to controls. In the Easy > Rest condition, this was observed in a region that extended from the right STG to the posterior insula and thalamus. In the Hard > Rest condition, very preterm participants showed decreased negative hemodynamic response compared to controls in the left and right STG (also extending to the insula) as well as the right sensorimotor cortex. In the Hard > Easy condition, very preterm adults showed greater hemodynamic response suppression compared to controls in bilateral LOC (Table 4; Fig. 4).

**Table 4.** Differences in hemodynamic responses between very preterm adults and controls during easy and hard letter trials

Condition	Region	Peak MNI coordinate [*x*,*y*,*z*] (mm)	Cluster size (voxels)	*p* value^*^	Contrast of parameter estimate (mean ± SD) (very preterm; control)
Easy > Rest					
Very preterm > control	Right STG, insula, thalamus	[68, –2, 4]	3838	<0.001	–2.65 ± 11.71; –12.39 ± 11.21
Hard > Rest					
Very preterm > control	Right STG, insula	[62, –18, –6]	8492	<0.001	0.02 ± 10.12; –11.56 ± 10.27
	Left STG, insula	[–54, –4, 2]	2079	0.02	–3.52 ± 13.56; –15.34 ± 10.86
	Right sensorimotor cortex	[48, –40, 68]	2013	0.02	–1.54 ± 14.16; –12.71 ± 13.99
Hard > Easy					
Very preterm < control	Left LOC	[–30, –76, 45]	2356	0.00567	–3.01 ± 19.21; 9.84 ± 16.43
	Right LOC	[43, –82, 30]	1944	0.0185	–2.33 ± 26.59; 6.34 ± 12.06

^*^Cluster *p* values were obtained with *z* = 2.3, *p* < 0.05 (corrected for family wise error rate across voxels).

STG = superior temporal gyrus; LOC = lateral occipital cortex.

The regions which displayed between-group differences in hemodynamic responses were also those that showed negative hemodynamic responses in both groups, with the exception of the thalamus, where positive hemodynamic response was found in the very preterm group. The hemodynamic responses in these regions ranges across negative and positive values in very preterm adults (Table 4).

### Tractography analysis

The AF and FAT did not differ between groups in terms of volume or HMOA in either hemisphere, nor did they differ in terms of LI.

### Functional-behavioral associations

The contrast of parameter estimates in regions where between-group differences in hemodynamic response where found (Easy > Rest: right STG; Hard > Rest: left STG, right STG, and right sensorimotor cortex, Hard > Easy: left and right LOC) was correlated with participants’ online task performance and head motion.

**Figure 5.**
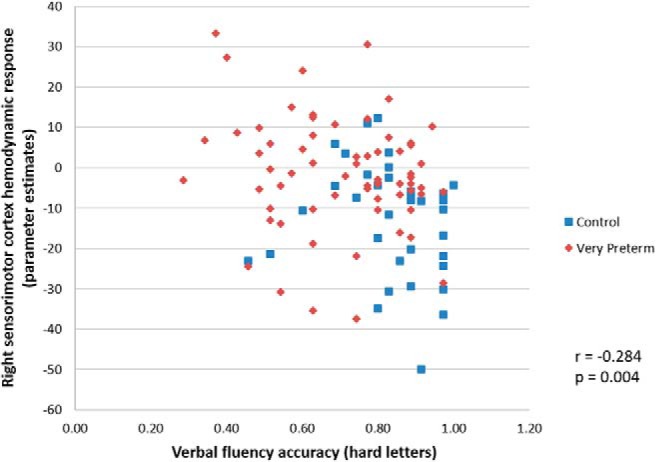
Verbal fluency accuracy and right sensorimotor cortex hemodynamic response during hard letter trials in the whole sample.

Only increased hemodynamic response in the right sensorimotor cortex in the Hard > Rest condition in the whole sample was significantly negatively correlated with performance on the hard letter trials of the on-line verbal fluency task (*r* = –0.284, *p* = 0.004), i.e., the greater the hemodynamic response the worse the performance (Fig. 5). All the correlation tests were corrected for multiple comparisons. Within-group analyses did not reveal any significant group-specific association between hemodynamic response and verbal fluency performance. Head motion during the fMRI task was not associated with any of the fMRI findings.

### Structural-behavioral associations

As no significant between-group differences in white matter tract indices were observed, associations between white matter tract indices and behavior were not further explored.

### Functional-structural associations

**Figure 6.**
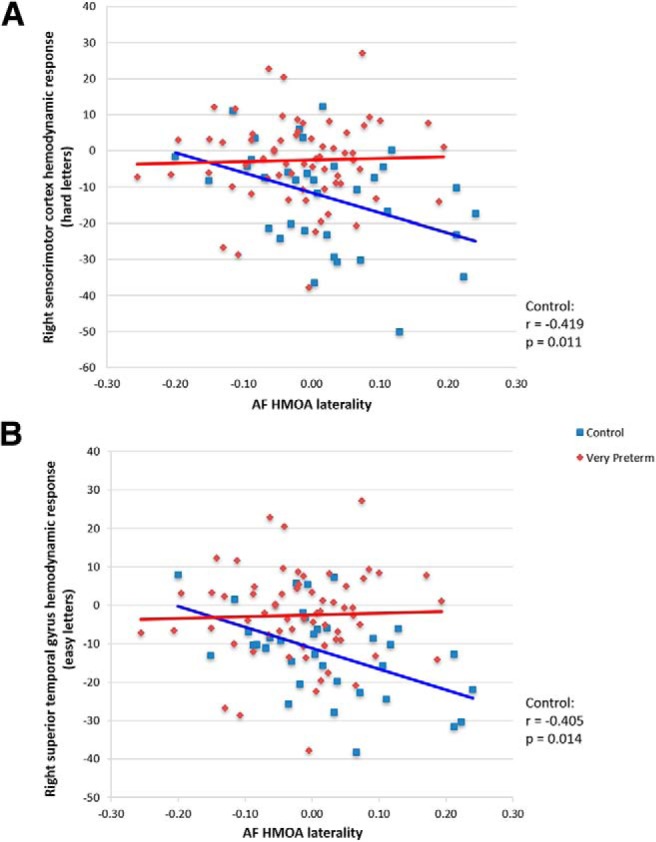
Associations between hemodynamic response and white matter characteristics in each group. ***A***, Right sensorimotor cortex hemodynamic response (hard letter trials) and AF HMOA laterality. ***B***, Right STG hemodynamic response (easy letter trials) and AF HMOA laterality. AF = arcuate fasciculus, HMOA = hindrance modulated orientational anisotropy.

Correlation tests across the whole sample did not show any significant functional-structural associations. Within-group analyses revealed group-specific patterns of association between hemodynamic response and white matter characteristics. Hemodynamic response in right sensorimotor cortex in the Hard > Rest condition significantly negatively correlated with the laterality of AF HMOA in controls (*r* = –0.419, *p* = 0.011), but not in very preterm individuals (*r* = 0.003, *p* = 0.981), i.e., the more hemodynamic response suppression the more left-lateralized the AF HMOA. This association was significantly different between groups (lateralization × group interaction: *F* = 7.446, *p* = 0.008; Fig. 6*A*). Hemodynamic response in the right STG in the Easy > Rest condition also significantly negatively correlated with AF HMOA laterality in controls (*r* = –0.405, *p* = 0.014), but not in very preterm individuals (*r* = 0.14, *p* = 0.269), and this significantly differed between groups (lateralization × group interaction: *F* = 5.494, *p* = 0.021; Fig. 6*B*). Hemodynamic response in the left STG in the Hard > Rest condition negatively correlated with the left FAT volume in the very preterm group and not in the control group, but this association was not significantly different between groups (volume × group interaction: *F* = 3.326, *p* = 0.071). All the correlation tests were corrected for multiple comparison correction.

### Analyses including only right-handed participants

As handedness may be associated with laterality (Knecht et al., 2000), all analyses were repeated for right-handed participants only (very preterm adults *n* = 52; controls *n* = 28). In these analyses, all significant results reported above remained unaltered, except for the association between left STG hemodynamic response during the hard letter trials and the left FAT volume in the very preterm group, which was no longer significant.

However, other significant structure-function associations became evident: in the very preterm group, but not in controls, higher left and right FAT HMOA were associated with increased right STG hemodynamic response suppression during hard letter trials (*r* = –0.411, *p* = 0.002; *r* = –0.315, *p* = 0.023). The association of the left FAT HMOA and right STG hemodynamic response was significantly different between groups (lateralization × group interaction: *F* = 4.44, *p* = 0.038).

### Association between verbal fluency and verbal IQ

In the whole sample, verbal IQ was significantly associated with verbal fluency performance on the easy and hard letter trials (*r* = 0.321, *p* = 0.002; *r* = 0.413, *p* < 0.001). Within-group analyses showed that verbal IQ was only significantly associated with verbal fluency on hard letter trials in very preterm adults (*r* = 0.42, *p* = 0.001) but not in controls (*r* = 0.296, *p* = 0.113). However, the difference between the correlation coefficients in the two groups was not statistically significant.

### Sex differences within the very preterm group

Very preterm males performed better than very preterm females on the easy letter trials (*U* = 301.0, *p* = 0.015); no sex difference was found on the hard letter trials (*U* = 389.0, *p* = 0.235). Very preterm males also had higher verbal IQ and performance IQ than very preterm females (verbal IQ: *U* = 214.5, *p* = 0.003, performance IQ: *U* = 239.0, *p* = 0.014). There was however no evidence of sex differences in regions where group differences in hemodynamic response were observed during verbal fluency processing.

## Discussion

This study investigated the functional and structural brain correlates of verbal fluency in adulthood following very preterm birth. At a functional level, results showed decreased hemodynamic response suppression in very preterm adults compared to controls in several brain regions, which seemed to be suboptimal for completion of hard letter verbal fluency trials. At a structural level, increased left-laterality in the AF was associated with increased right hemispheric functional deactivation in controls but not in very preterm adults. These findings suggest that alterations in the typical development of left-lateralization in very preterm individuals are still present in adulthood.

### fMRI results and verbal fluency performance

Very preterm adults compared to controls showed decreased hemodynamic response suppression in the right STG, posterior insula and thalamus during completion of both easy and hard letters of a verbal fluency task. During processing of hard letters, altered hemodynamic responses in the very preterm group were more extensive and included left STG and insula and right sensorimotor cortex. Hemodynamic responses in these regions showed a more dynamic range of both positive and negative measures in very preterm adults. This could reflect individual differences when performing verbal fluency, with some participants engaging regions that are not typically required for the specific tasks or some participants failing to suppress a region. Taken together with the findings that very preterm adults performed worse on the hard letters but not the easy letters compared to term-born controls, these results suggest that hemodynamic responses are particularly affected when a task presents high-cognitive demands. In the following paragraphs we will discuss findings with regards to each region.

The STG has been recognized to play a role in speech recognition and comprehension. The left and right hemisphere, however, process speech differently. Hickok and Poeppel (2007) proposed that integration of information over longer timescales predominantly occurs in the right hemisphere, while integration over shorter timescales may be more bilateral. Another view is that the left hemisphere may be associated with phonemic perception and process information more categorically than the right hemisphere (Liebenthal et al., 2005). Other than differences in speech processing, the left and right STG also differ in their involvement in speech production. Specifically, the left posterior STG is suggested to be involved in the phonological processing of both speech input and output (Hickok et al., 2003, 2009). In regards to verbal fluency, a previous PET study revealed a decrease in relative cerebral blood flow in bilateral STG during a letter verbal fluency task in controls (Frith et al., 1991). Similarly, decreased hemodynamic response was found in the right STG when comparing hemodynamic response during verbal fluency to an automatic speech control condition in healthy participants (Birn et al., 2010). These differences could be due to differences in auditory processing and STG suppression may be needed to perform the task.

The insula has a known role in language processing due to its strong connections to the IFG and temporal cortex. In particular, the posterior insula has been found to be involved in word retrieval and lexical knowledge (Ardila et al., 2014), which is utilized during verbal fluency tasks. Based on a model proposed by Just and Varma (2007), when a task is sufficiently difficult, resource demands on the typical brain network engaged by such task will exceed resource supplies, and additional brain regions with spare resources and relevant functional specializations will be recruited to aid task performance. When an individual’s resource supply is reduced as a result of neurodevelopmental alterations, recruitment of additional brain regions to aid task performance may occur. It was previously shown that individuals born very preterm who sustained perinatal brain injury displayed increased hemodynamic response in bilateral insula and associated perisylvian areas, and this correlated with performance on a verbal working memory task (Froudist-Walsh et al., 2015). The insula is also involved in a wide range of other functions, such as auditory, motor, affective and gustatory processing (Chang et al., 2013). Very preterm adults may have showed decreased hemodynamic response suppression in the insula during completion of a verbal fluency task because they may have required the support of a wider range of cognitive functions than those employed by control participants. The “extra” recruitment of hemodynamic resources during language processing has been previously observed in preterm adolescents during performance of a sentence comprehension task (Barde et al., 2012).

Increased hemodynamic response in the very preterm compared to the control group was also found in the thalamus. The thalamus is activated during letter fluency in healthy controls (Ravnkilde et al., 2002), and thalamic lesions lead to impairment in verbal fluency (Annoni et al., 2003). The thalamus is vulnerable to very preterm birth and volumetric deficits are often described in very preterm individuals (Boardman et al., 2006; Nosarti et al., 2014). Volumetric reductions of the thalamic nuclei have been associated with worse letter verbal fluency in very preterm adolescents (Gimenez et al., 2006). The thalamus may represent a central monitor for language-related cortical activities, controlling and adapting the connectivity between cortical regions and bandwidth the exchange of information (Klostermann et al., 2013). The increased hemodynamic response in the thalamus we see in our results may indicate the increased effort very preterm adults need to complete a letter fluency task, although we only noticed this during the easy and not the hard letters. It is therefore possible that increased thalamic response is reflective of more effective information processing to facilitate task performance.

The sensorimotor cortex was the only region that showed decreased hemodynamic response suppression during completion of hard letter trials in the preterm group compared to controls that is not typically involved in language processing. The cortical systems for action control and language were traditionally thought to be independent systems, although more recent theoretical views suggest these may be served by interactive functional systems (Pulvermuller, 2005). Evidence of white matter connections between motor and language regions and somatotopic activation in the motor cortex in response to action-related words supports this notion (Pulvermuller, 2005; Pulvermuller and Fadiga, 2010). Schafer et al. (2009) found that in preterm adolescents, hemodynamic response in the left sensorimotor cortex during a lexical semantic association fMRI task was correlated with better task performance. In the same study, functional connectivity between typical language-related temporal and sensorimotor areas was only present in preterm adolescents, suggesting that the sensorimotor cortex may mediate connections between language areas in the preterm brain.

Using a verbal fluency task, we found that at the whole group level decreased hemodynamic response suppression in right sensorimotor cortex during completion of the hard letter trials was associated with participants’ poorer task performance, supporting the idea that increased neural recruitment does not necessarily lead to better cognitive performance (Turkeltaub et al., 2012; Tseng et al., 2017). This finding may be expected given that significant group differences in verbal fluency (hard letters) and right sensorimotor cortex hemodynamic response were found. Nonetheless, other regions that also exhibited differences in hemodynamic response did not show an association with verbal fluency performance. Previous research suggested that recruitment of right hemispheric mechanisms for language may occur when left hemispheric specialization is disrupted, although it is unclear whether this leads to the successful acquisition of typical language skills (Holland et al., 2007). Contrasting findings between the current and Schafer’s study could be due to the use of different tasks assessing different language processes.

Around half of all participants (and the majority of controls) had a negative contrast of parameter estimate in the right sensorimotor cortex, indicating that suppression of this region compared to the baseline is needed to perform well on a verbal fluency task. Intrasubject comparisons of fMRI deactivation during visual attention and working memory processing suggest that deactivation may be an inhibition mechanism to reduce distracting neural processes, rather than a local reduction of relative cerebral blood flow in less active brain regions due to increased relative cerebral blood flow in activated brain regions (Tomasi et al., 2006). Better visual attention performance has in fact been associated with stronger disconnection of task-irrelevant brain regions (Tomasi et al., 2014).

Greater LOC hemodynamic response suppression in very preterm adults compared to controls in the Hard > Easy condition could be related to differences in word form processing. The LOC is connected to the visual word form area through the vertical occipital fasciculus (Yeatman et al., 2013). Damage to the anterior vertical occipital fasciculus has been found to impair reading abilities (Yeatman et al., 2014). It is possible that this region is more engaged during the REST control condition when reading a word and dependent on successful word retrieval during the task conditions. However, white matter properties and task performance were not associated with this difference.

### Structural MRI results

Contrary to our prediction, very preterm adults did not have smaller volume and HMOA and decreased-left lateralization in both structural indices of the AF and FAT compared to term-born controls. One possible explanation could be that the primary site of perinatal injury (i.e., periventricular hemorrhage) involves periventricular regions, therefore affecting subcortical regions and its connections (e.g., the dorsal and ventral cingulum and the fornix) to a greater extent than structures that lie more laterally in the brain (Froudist-Walsh et al., 2015). In previous studies, it was also shown that the superior longitudinal fasciculus, which is distant from the ventricles, did not exhibit between-group volumetric differences, suggesting that there may be a medial-lateral gradient of risk for structural injury following very preterm birth (Froudist-Walsh et al., 2015; Caldinelli et al., 2017). A lack of significant group differences in AF and FAT, which connect to or within the frontal lobe, could be also interpreted using a neurodevelopmental perspective: the frontal lobe displays protracted maturation compared to other brain areas (Petanjek et al., 2011), possibly resulting in decreased vulnerability of its white matter connections to early brain insults.

### Functional-structural associations

We expected that increased right hemispheric hemodynamic response in very preterm adults would be associated with increased right-lateralization of AF or FAT white matter indices. Instead, only in controls we found an association between increased right-lateralization of AF HMOA and decreased hemodynamic response suppression in right STG in the Easy > Rest condition and in right sensorimotor cortex in the Hard > Rest condition. As decreased hemodynamic response suppression in right sensorimotor cortex was associated with worse verbal fluency performance on hard letter trials, these findings highlight the importance of left-lateralization for language-related functions. Part of the left AF is considered as a direct phonologic pathway and may be particularly important to aid children’s language acquisition (Glasser and Rilling, 2008), and early leftward AF asymmetry is seen in term-born infants (Dubois et al., 2009). The fact that this was not found in very preterm adults may indicate a lateralization alteration, considering that the asymmetry of the cerebral hemispheres (most prominently in perisylvian cortex) emerges during the late second and third trimester of gestation, when very preterm birth occurs (Habas et al., 2012).

Neuroimaging studies investigating language functions in preterm individuals have highlighted the importance of interhemispheric connections and lateralization in language development (Salvan and Nosarti, 2018). An increased right-hemispheric engagement found in this study has been previously reported during language tasks in preterm individuals (Gozzo et al., 2009; Myers et al., 2010; Scheinost et al., 2015) and may reflect deviations in typical cortical language network development, when functional specialization increases (Skeide and Friederici, 2016). Atypical lateralization of language networks has also been shown in disorders such as autism spectrum disorder and schizophrenia (Mitchell and Crow, 2005; Preslar et al., 2014). We speculate that the atypical functional lateralization of verbal fluency networks seen here could contribute to the increased psychiatric risk in very preterm samples (Nosarti et al., 2012).

While not demonstrating a significant association between right STG and right sensorimotor cortex hemodynamic response and the AF seen in controls, very preterm adults instead showed a distinct relationship between increased right STG hemodynamic response suppression during hard letter trials and higher left FAT HMOA. This finding is consistent with other studies proposing that the FAT plays a role in verbal fluency processing in clinical populations (Catani et al., 2013; Kronfeld-Duenias et al., 2016). Together with the previously discussed findings, our results suggest a remapping of the neuroanatomical underpinnings of verbal fluency to prioritize the left FAT in very preterm adults. However, as neither left FAT HMOA nor right STG hemodynamic response showed a significant association with on-line task performance, with the current results we are unable to determine if this observed structural-functional association may be adaptive or maladaptive. Another interpretation for our unique within-group results could be that the two tracts we investigated, the AF and the FAT, which are differentially involved in various aspects of language (Catani and Bambini, 2014), may be supporting distinct linguistic operations in controls and very preterm adults. It was not within the scope of this study to carry out an extensive assessment of language processing and further studies are needed to pinpoint the specific functions of each tract in typically and atypically developing samples.

### Brain lateralization and language

So far in the reviewed literature, left-lateralization of the brain has been associated with better language skills. However, previous studies have also reported no relationship between functional brain lateralization and language skills in healthy subjects (Knecht et al., 2001), but in those with developmental difficulties (Illingworth and Bishop, 2009). It is possible that atypical cerebral lateralization is a potential risk factor for language impairment and the addition of or interaction with other factors (e.g., genetic) may be the cause of language difficulties (Bishop, 2013). It is worth highlighting that cerebral lateralization can change throughout development and may be a consequence rather than a cause of poor language abilities (Bishop, 2013).

### Sex differences within the very preterm group

Contrary to previous findings that preterm girls outperform boys on language skills (Eriksson et al., 2012), this study found that very preterm men performed better than women on the easy letters during the verbal fluency task and had higher verbal IQ. However, in the larger sample the current participants were drawn from (Kroll et al., 2017), there were no sex differences in verbal IQ. Future studies with larger sample sizes are needed to confirm whether there are sex differences in language abilities in very preterm adults.

### Limitations

We acknowledge that there are several limitations to this study. The nature of verbal fluency, being a combined measure of verbal and executive function abilities, makes it difficult to tease out which cognitive component may be affected in a specific population sample. This study selectively focused on the language component of the task. The executive function component of verbal fluency and corresponding white matter connections, which may explain other aspects of the long-term sequelae of very preterm birth, remains an area to explore further.

Very preterm adults in this study only showed lower verbal and not performance IQ compared to controls, although in the larger sample they were drawn from, they had lower verbal and performance IQ (Kroll et al., 2017). In this study, we found that poorer verbal IQ was associated with worse verbal fluency on the hard letter trials in the very preterm group only, suggesting that verbal fluency may represent one of the various aspects of language processing that may be affected by very preterm birth, although not assessed here.

There are a number of potential methodological limitations. First, is the exclusive consideration of white matter fiber tracts that we thought to be involved in verbal fluency. Therefore, we did not investigate other tracts, such the uncinate fasciculus, which enables the mapping of sound to meaning and is viewed as a critical component of the language network (Friederici and Gierhan, 2013), yet has not been directly implicated in letter fluency (Catani et al., 2013; Kljajevic et al., 2016). Second, there is a concern that false positive rates of fMRI findings using parametric statistical methods with cluster-based inference is higher than anticipated (Eklund et al., 2016). There is currently no non-parametric equivalent of FEAT’s FLAME to assess differences in findings between parametric and nonparametric methods. Therefore, the results reported in this study should be interpreted with caution and future work to validate these findings with non-parametric methods is needed.

## Conclusion

Very preterm adults exhibited worse verbal fluency performance than controls when a high cognitive demand was required. The results of this study suggest that this may be due to deviations in typical development, resulting in a less left-lateralized network underlying verbal fluency. Verbal fluency processing in very preterm adults may be supported by a potential remapping of structural-functional brain associations, involving the FAT. Based on this study, future work is warranted to explore the development of brain lateralization in very preterm individuals at different stages of maturation.

## Synthesis

Reviewing Editor: Morgan Barense, University of Toronto Decisions are customarily a result of the Reviewing Editor and the peer reviewers coming together and discussing their recommendations until a consensus is reached. When revisions are invited, a fact-based synthesis statement explaining their decision and outlining what is needed to prepare a revision will be listed below. The following reviewer(s) agreed to reveal their identity: Margot Taylor, Annika Linke.

We all agreed that the manuscript was well written and the study was well motivated. Moreover, combining task fMRI and diffusion weighted imaging is a powerful way to address how very premature birth impacts brain function and structure in the context of verbal fluency. However, both reviewers raised some concerns and questions, which are detailed below. We hope that you can address these in a revision.

Reviewer 1:

This paper is looking at fMRI during two conditions in a verbal fluency task as well as measuring two white matter tracts proposed to be related to verbal fluency in adults who were or were not born very preterm. I have a number of concerns about some of the methodological details in this paper and a number of suggestions for the authors.

In the abstract, I don't think that 'human' needs to be mentioned, as studying verbal fluency in other than humans would be quite difficult. At the end of the abstract, and the same for the significance statement, it would be better to be worded so that it is more accessible. Saying that there is an association is not as helpful is saying that there was greater bilateral representation in the very preterm group, etc., for example.

The authors referred to HMOA in the introduction without defining or describing it; both need to be done.

In the methods section, it would good to include in the text if the subjects were matched on sex and their ages. In figure 1 for the methods, it would be helpful to have some information about the timing - how long the trials and blocks lasted. The authors say that the maximum B value was 1300 suggesting that the B values were variable; if this is the case what was the range and mean? However, the authors use constrained spherical deconvolution and that pipeline typically requires much higher b-values; can they explain this? They also give no details about their motion correction, were there were differences between the groups? How did they correct for motion in their analyses? What about slice-timing correction?

It seems as though the task was run as a block design but if only correct responses were used, were subsets of the blocks analysed? They are following the protocol first used by Fu et al (2002) yet they do not follow the analysis procedures of Fu. They do not compare the easy versus hard conditions. This should be done and the data on the easy vs. hard would also remove the issues discussed in their limitations section about the resting state deactivation impacting the results.

Why do they refer to normalising to a very preterm born adult template, which would bias towards the preterm adults, and then say that the template included both preterm and term born individuals?

Again in the DTI analyses they do not say what they used for motion correction -this needs to be detailed. They also need to better explain their use of the FOD parameters that were used (p. 11, lines 15 and 16) as the methods were not clear and are different from what is recommended on the related websites I could find.

Also according to the guidelines for the fibre orientation streamlines, they likely should not have exceeded 40{degree sign} (line 21, page 11); how do they justify using 60{degree sign}?

Their figure 2 of their ROIs was disconcerting; they are marking their ROIs on a very degraded image. Would it not have been better to use the FA image or a mean DTI image and be able to draw more specific ROIs? Also, it shows a large area as the IFG which seems much larger than what would typically be referred to as the IFG. Please state the parameters used to define it, as is normally should be BA 45 and 44, for example.

The point about testing for normality was repeated pages 12 and 13/14.

The authors ran many functional-structural correlations; I wondered if they were corrected for all the multiple comparisons. They report ones that were close or significant but I imagine others were run as well.

Table 6 has only one measure in one group that survives correction; therefore the table does not need to be part of the main text. Again, it should also be a comparison between the easy and hard conditions and not the easy and rest, and hard and rest.

I think the second sentence in the discussion is an overstatement as the very preterm adults were still able to do the verbal fluency task at the higher level but simply had more errors; it was not as though they could not do it or that the brain network was not able to support task performance.

On page 29 the authors refer to preterm adults recruiting additional brain regions 'earlier'; far as I could tell there was no timing information in any of their measures. Please re-work the section.

The discussion of the thalamus activation in on p. 30 needs to include the point that it was only in the easy task, therefore why would it be involved only in the simple task where there were no behavioral differences and not in the harder task?

The authors conclude that there is atypical structural lateralization (p. 34) when in fact they did not find structural differences in the two fiber tracts that they analyzed - this needs to be modulated.

Reviewer 2:

The manuscript “Verbal fluency is affected by altered brain lateralisation in adults who were born very preterm” is well written and the study is well motivated. The main hypotheses clearly follow from a thorough review of the previous literature. Combining task fMRI and diffusion weighted imaging is a powerful way to address how very premature birth impacts brain function and structure in the context of verbal fluency. Results of all analyses are presented in detail. There are a few conceptual and methodological questions, however, that remain, and that should be addressed in a revision of this manuscript before I can recommend it for publication:

1. The main interpretation of the results presented is that brain lateralization is reduced in adults who were born very prematurely compared to a control group, and that this “increased neural recruitment” of regions in the right hemisphere is related to worse performance on the verbal fluency task. This conclusion is drawn because parameter estimates for the contrasts of interest are significantly higher in the right STG and right sensorimotor cortex in adults born prematurely. The authors present average parameter estimates for these contrasts for both groups, and note themselves that these were negative for both groups. The significant difference between the preterm and control group results from stronger deactivation in the control group. It would be helpful to also show significant deactivations in Figure 3 and Table 2. In any case, given the parameter estimates presented in Table 4, it is likely that deactivation would only be significant in the control group. Rather than interpreting this finding as showing “increased neural recruitment” (and reduced lateralization) in the preterm group, it would be more appropriate to discuss the absence of deactivation (which might reflect a lack of suppression rather than increased neural recruitment) in the preterm group. The discussion and interpretation of the results should be revised with this in mind.

2. The description of the task paradigm and how it was modeled would benefit from additional details. Most importantly, no stimulus/trial/block timings are given. How long was each letter presented for, and how much time did participants have to respond? How were the different conditions and individual letters randomized? How was response duration/time and correct/incorrect responses modeled in the GLM?

3. Only task accuracy is summarized and analyzed. It would be interesting to also see average response times for both groups. Did it take the preterm group significantly longer to produce (correct) responses? If so, is response time related to differences in sensorimotor deactivation or any of the other results?

4. Given participants were required to overtly generate words, does the amount of motion correlate with any of the main findings (and particularly with differences in sensorimotor deactivation)?

5. Why did the authors only model Easy and Hard conditions against rest, but did not contrast the Easy and Hard conditions directly?

6. The authors are probably aware of the “Cluster failure” debacle (http://www.pnas.org/content/113/28/7900). Would the results presented survive permutation testing (e.g. using RANDOMISE in FSL)?

7. The authors mention excluding one participant from the tractography analysis due to poor data quality. What quality control and/or motion correction was performed on the DWI data?

8. The whole sample correlation between verbal fluency accuracy (for hard letters) and right sensorimotor cortex activation is to be expected given significant group differences in both verbal fluency accuracy (hard letters) and right sensorimotor cortex activation had already been shown. This should be acknowledged when discussing this finding. It would be helpful if data points in Figure 5 were color coded by group even if the correlation is shown for the whole sample, e.g. when the authors state that “Around half of all participants (and the majority of controls) had negative contrast of parameter estimate in the right sensorimotor cortex” (pg. 31).

9. The interpretation that “the greater the hemodynamic response the less left-lateralised the AF HMOA” (pg. 24) is misleading given that greater hemodynamic response here means less deactivation (see comment 1 above).

10. Slightly more boys than girls are born prematurely (https://www.nature.com/articles/pr2013204), girls born prematurely often have a better prognosis (https://www.ncbi.nlm.nih.gov/pubmed/22184652), and girls tend to outperform boys on language skills (https://www.ncbi.nlm.nih.gov/pubmed/22550951). As such it would be very interesting to analyze this study's results for sex differences.

Minor concerns/suggestions

1. This is personal preference and the authors may ignore it, but I would prefer if participant demographics were already summarized (and Table 1 referred to) in the Methods and Materials section rather than presenting this information in the Results section.

2. Which demographic and behavioral data was used for behavioral correlations should be stated clearly in the “Statistical analysis of demographic and behavioral data and integration of imaging-derived measures” section (pg. 13).

3. Which multiple comparison correction and (cluster-correction?) thresholds were used for Table 2, Figure 3, Table 3, and Figure 4 should be mentioned in respective captions. Labels should be added to the color bars shown in the Figures.

## Author Response

Synthesis of Reviews:

Computational Neuroscience Model Code Accessibility Comments for Author (Required):

N/A

Significance Statement Comments for Author (Required):

In general, this statement should be made more accessible to non-specialists. For example, it would be helpful to state explicitly why these findings are important, as well as some brief orienting comment(s) about the specific brain networks/tracts mentioned.

Comments on the Visual Abstract for Author (Required):

N/A

Synthesis Statement for Author (Required):

We all agreed that the manuscript was well written and the study was well motivated. Moreover, combining task fMRI and diffusion weighted imaging is a powerful way to address how very premature birth impacts brain function and structure in the context of verbal fluency. However, both reviewers raised some concerns and questions, which are detailed below. We hope that you can address these in a revision.

Reviewer 1:

This paper is looking at fMRI during two conditions in a verbal fluency task as well as measuring two white matter tracts proposed to be related to verbal fluency in adults who were or were not born very preterm. I have a number of concerns about some of the methodological details in this paper and a number of suggestions for the authors.

-In the abstract, I don't think that 'human' needs to be mentioned, as studying verbal fluency in other than humans would be quite difficult. At the end of the abstract, and the same for the significance statement, it would be better to be worded so that it is more accessible. Saying that there is an

association is not as helpful is saying that there was greater bilateral representation in the very preterm group, etc., for example.

Reply: We thank the reviewer for this comment. The end of the abstract and the significance statement have been revised.

-The authors referred to HMOA in the introduction without defining or describing it; both need to be done.

Reply: We thank the reviewer for this comment. HMOA is now defined and described on page 5 lines 78.

-In the methods section, it would good to include in the text if the subjects were matched on sex and their ages. In figure 1 for the methods, it would be helpful to have some information about the timing - how long the trials and blocks lasted. The authors say that the maximum B value was 1300 suggesting that the B values were variable; if this is the case what was the range and mean? However, the authors use constrained spherical deconvolution and that pipeline typically requires much higher b-values; can they explain this? They also give no details about their motion correction, were there were differences between the groups? How did they correct for motion in their analyses? What about slice-timing correction?

Reply: We thank the reviewer for this comment. We aimed to match control participants in age; this is added on page 6 line 5-6. There were no group differences in sex and age as shown in Table 1.

The timing information for the fMRI task is added on page 8 lines 7-15.

The diffusion imaging sequence was single shell and there was only one b value, this has been rewritten on page 9 line 14.

We chose this approach as tractography using constrained spherical deconvolution outperforms tractography using other reconstruction methods when using data acquired with clinical b-values (Wilkins et al., 2015). This is stated on page 12, lines 11-14.

Motion correction was done using FSL's FLIRT for the fMRI data, this is added on page 10 line 1-2. There were no group differences in motion in both the diffusion data and fMRI data, this information is added on page 10 lines 3-5 and page 12 lines 7-9. Slice-timing correction was performed in the fMRI data; this is added on page 10 line 2.

-It seems as though the task was run as a block design but if only correct responses were used, were subsets of the blocks analysed? They are following the protocol first used by Fu et al (2002) yet they do not follow the analysis procedures of Fu. They do not compare the easy versus hard conditions. This should be done and the data on the easy vs. hard would also remove the issues discussed in their limitations section about the resting state deactivation impacting the results.

Reply: We thank the reviewer for this comment. Although the VF task was a block design in the analyses we only modeled the timing of the events associated with successful performance and not the whole block. This was done in order to control for the potentially confounding effects of differences in task performance on BOLD signal.

The findings from the Hard &gt; Easy contrast are added in Results on page 21 lines 1-4, Table 4, and Figure 4.

-Why do they refer to normalising to a very preterm born adult template, which would bias towards the preterm adults, and then say that the template included both preterm and term born individuals?

Reply: We thank the reviewer for this comment. The template used included both very preterm and term born individuals, the description of the template has been changed to a study-specific template on page 11 line 21.

-Again in the DTI analyses they do not say what they used for motion correction -this needs to be detailed. They also need to better explain their use of the FOD parameters that were used (p. 11, lines 15 and 16) as the methods were not clear and are different from what is recommended on the related websites I could find.

Reply: Motion correction was done using the motion and eddy current correction tool in ExploreDTI, this is stated on page 12 lines 4-7.

The FOD parameters used in this study are the same as those used in previously published work (Froudist-Walsh et al., 2015; Karolis et al., 2016; Tseng et al., 2017). Visual inspection was performed in regions with known crossing fibres (e.g. between the corpus callosum, superior longitudinal fasciculus, and corticospinal tract) and without (e.g. middle of the corpus callosum). This is added on page 12 lines 21-25. An example of the reconstructed fiber orientation distributions using the stated tractography reconstruction parameters is shown in Figure R1.

Figure R1. An example of the reconstructed fibre orientation distributions using the chosen parameters. The crossing fibres between the corpus callosum (red), corona radiata (blue), and superior longitudinal fasciculus (green) can be resolved. Less anisotropic voxels are removed by the absolute threshold (red ball) from further analysis.

-Also according to the guidelines for the fibre orientation streamlines, they likely should not have exceeded 40{degree sign} (line 21, page 11); how do they justify using 60{degree sign}?

Reply: One of the tracts that we targeted, the arcuate fasciculus, is a curved bundle. Therefore, we used a more lenient angular threshold to ensure the arcuate fasciculus could be reconstructed in all participants. This threshold is also close to that used by Phillips et al., (55 degrees) to preclude the generation of fibers with biologically unrealistic curvature (i.e., “looping” fibers) (Phillips et al., 2012). This is added on page 13 lines 9-13. Artefactual/non-anatomical tracts were manually removed based on the literature of brain anatomy and shape of the tracts (Catani et al., 2012; Dick and Tremblay, 2012), as stated on page 14 lines 2-4.

-Their figure 2 of their ROIs was disconcerting; they are marking their ROIs on a very degraded image. Would it not have been better to use the FA image or a mean DTI image and be able to draw more

specific ROIs? Also, it shows a large area as the IFG which seems much larger than what would typically be referred to as the IFG. Please state the parameters used to define it, as is normally should be BA 45 and 44, for example.

Reply: We thank the reviewer for this comment. The presented image was on the FA image which was at a resolution of 2.4x2.4x2.4mm, though it looked particularly granular. We created a new figure using a different visualization software. The IFG was defined as BA45 and 44, this description is added on page 13 line 25. It seems larger on the image because it is shown on a more lateral sagittal slice of the brain. We decided to change Figure 2 to show an example of the dissected tracts instead of the figure of ROIs.

Figure R2. An example of the region-of-interest approach used for tractography.

-The point about testing for normality was repeated pages 12 and 13/14.

Reply: We thank the reviewer for this comment. We have deleted this information.

-The authors ran many functional-structural correlations; I wondered if they were corrected for all the multiple comparisons. They report ones that were close or significant but I imagine others were run as well.

Reply: We thank the reviewer for this comment. Yes, all the results were corrected for multiple comparisons. This is added on page 25 lines 12-13.

-Table 6 has only one measure in one group that survives correction; therefore the table does not need to be part of the main text. Again, it should also be a comparison between the easy and hard conditions and not the easy and rest, and hard and rest.

Reply: We thank the reviewer for this comment. This table has been removed from the main text.

-I think the second sentence in the discussion is an overstatement as the very preterm adults were still able to do the verbal fluency task at the higher level but simply had more errors; it was not as though they could not do it or that the brain network was not able to support task performance.

Reply: We thank the reviewer for this comment. This sentence has been reworded on page 29 lines 3-6.

-On page 29 the authors refer to preterm adults recruiting additional brain regions 'earlier'; far as I could tell there was no timing information in any of their measures. Please re-work the section.

Reply: We thank the reviewer for this comment. This sentence has been reworded on page 31 lines 4-6.

-The discussion of the thalamus activation in on p. 30 needs to include the point that it was only in the easy task, therefore why would it be involved only in the simple task where there were no behavioral differences and not in the harder task?

Reply: We thank the reviewer for this comment. This is now discussed in Discussion page 32 lines 2-7.

-The authors conclude that there is atypical structural lateralization (p. 34) when in fact they did not find structural differences in the two fiber tracts that they analyzed - this needs to be moderated.

Reply: We thank the reviewer for this comment. This sentence has been reworded on page 36 line 2.

Reviewer 2:

The manuscript “Verbal fluency is affected by altered brain lateralisation in adults who were born very preterm” is well written and the study is well motivated. The main hypotheses clearly follow from a thorough review of the previous literature. Combining task fMRI and diffusion weighted imaging is a powerful way to address how very premature birth impacts brain function and structure in the context of verbal fluency. Results of all analyses are presented in detail. There are a few conceptual and methodological questions, however, that remain, and that should be addressed in a revision of this manuscript before I can recommend it for publication:

1. The main interpretation of the results presented is that brain lateralization is reduced in adults who were born very prematurely compared to a control group, and that this “increased neural recruitment” of regions in the right hemisphere is related to worse performance on the verbal fluency task. This conclusion is drawn because parameter estimates for the contrasts of interest are significantly higher in the right STG and right sensorimotor cortex in adults born prematurely. The authors present average parameter estimates for these contrasts for both groups, and note themselves that these were negative for both groups. The significant difference between the preterm and control group results from stronger

deactivation in the control group. It would be helpful to also show significant deactivations in Figure 3 and Table 2. In any case, given the parameter estimates presented in Table 4, it is likely that deactivation would only be significant in the control group. Rather than interpreting this finding as showing “increased neural recruitment” (and reduced lateralization) in the preterm group, it would be more appropriate to discuss the absence of deactivation (which might reflect a lack of suppression rather than increased neural recruitment) in the preterm group. The discussion and interpretation of the results should be revised with this in mind.

Reply: We thank the reviewer for this comment. Significant negative hemodynamic responses have been added to Figure 3 and Table 3. The interpretation of the results has been revised in Discussion.

2. The description of the task paradigm and how it was modeled would benefit from additional details. Most importantly, no stimulus/trial/block timings are given. How long was each letter presented for, and how much time did participants have to respond? How were the different conditions and individual letters randomized? How was response duration/time and correct/incorrect responses modeled in the GLM?

Reply: We thank the reviewer for this comment. The timing information for the fMRI task is added on page 8 lines 7-15. The letters were presented in the same order for all participants. The responses were modeled as trials of 4 seconds duration (1 inter-stimulus interval) and only corrected responses were given a value (1) in the model.

3. Only task accuracy is summarized and analyzed. It would be interesting to also see average response times for both groups. Did it take the preterm group significantly longer to produce (correct) responses? If so, is response time related to differences in sensorimotor deactivation or any of the other results?

Reply: We thank the reviewer for this comment. The average correct response time was not different between groups for both easy and hard letters. This is added in Results page 16 lines 5-7 and Table 2.

4. Given participants were required to overtly generate words, does the amount of motion correlate with any of the main findings (and particularly with differences in sensorimotor deactivation)?

Reply: We thank the reviewer for this comment. No association between motion and fMRI findings or task performance was found. This is added on page 23 lines 22-23.

5. Why did the authors only model Easy and Hard conditions against rest, but did not contrast the Easy and Hard conditions directly?

Reply: We thank the reviewer for this comment. The Hard &gt; Easy contrast has been added in Results page 21 lines 1-4, Table 4, and Figure 4.

6. The authors are probably aware of the “Cluster failure” debacle (http://www.pnas.org/content/113/28/7900). Would the results presented survive permutation testing (e.g. using RANDOMISE in FSL)?

Reply: We thank the reviewer for this comment. When running the same analysis using randomise with 1000 permutations, group differences were only found in the right STG during Hard &gt; Rest, in a region similar to that found in the original FLAME analysis results (Figure R3). Differences in the results from randomise and FLAME can be expected because they estimate mixed effect variances differently (randomise is ordinary least-squares based, which does not consider within subject variance when estimating mixed effect variances the way FLAME does) and thus cannot be compared. There is currently no non-parametric equivalent of FLAME to assess differences in findings between parametric and nonparametric methods. However, we acknowledge the rate of false positive may be higher than expected in our findings and future work to validate these findings with non-parametric methods is needed. This has been added as a limitation of the study on page 38 lines 18-24.

Figure R3. Group differences during hard letter trials using FLAME (blue) and randomize (green).

7. The authors mention excluding one participant from the tractography analysis due to poor data quality. What quality control and/or motion correction was performed on the DWI data?

Reply: We thank the reviewer for this comment. At the time of the analysis, we were unable to reconstruct tracts for one control participant and thought this was due to data quality after visual inspection. After revisiting the data, we are now able to reconstruct this participant's tracts and relevant results have been revised. ExploreDTI's motion and eddy current correction was performed for all participants, this is stated on page 12 lines 4-7. The final reconstructed whole-brain tractography was visually assessed. This is added on page 13 and lines 14-15.

8. The whole sample correlation between verbal fluency accuracy (for hard letters) and right sensorimotor cortex activation is to be expected given significant group differences in both verbal fluency accuracy (hard letters) and right sensorimotor cortex activation had already been shown. This should be acknowledged when discussing this finding. It would be helpful if data points in Figure 5 were color coded by group even if the correlation is shown for the whole sample, e.g. when the authors state that “Around half of all participants (and the majority of controls) had negative contrast of parameter estimate in the right sensorimotor cortex” (pg. 31).

Reply: We thank the reviewer for this comment. The discussion of the whole sample correlation between verbal fluency accuracy (for hard letters) and right sensorimotor cortex activation has been expanded in Discussion on page 33 lines 2-6. Figure 5 has been changed to color code the groups.

9. The interpretation that “the greater the hemodynamic response the less left-lateralised the AF HMOA” (pg. 24) is misleading given that greater hemodynamic response here means less deactivation (see comment 1 above).

Reply: We thank the reviewer for this comment. The interpretation of the findings has been revised in Discussion.

10. Slightly more boys than girls are born prematurely (https://www.nature.com/articles/pr2013204), girls born prematurely often have a better prognosis (https://www.ncbi.nlm.nih.gov/pubmed/22184652), and girls tend to outperform boys on language skills (https://www.ncbi.nlm.nih.gov/pubmed/22550951). As such it would be very interesting to analyze this study's results for sex differences.

Reply: We thank the reviewer for this comment. We found that very preterm men performed better than women on the easy letters during the verbal fluency task. They also had higher verbal and performance IQ. These results are added in Results page 27 lines 23-25 and page 28 lines 1-5; and discussed in Discussion page 37 lines 13-20.

Minor concerns/suggestions

1. This is personal preference and the authors may ignore it, but I would prefer if participant demographics were already summarized (and Table 1 referred to) in the Methods and Materials section rather than presenting this information in the Results section.

Reply: We thank the reviewer for this comment. Information of participant demographics has been moved to the Methods and Materials section.

2. Which demographic and behavioral data was used for behavioral correlations should be stated clearly in the “Statistical analysis of demographic and behavioral data and integration of imaging-derived measures” section (pg. 13).

Reply: We thank the reviewer for this comment. The demographic and behavioral data are stated in Methods page 15 lines 6-8.

3. Which multiple comparison correction and (cluster-correction?) thresholds were used for Table 2, Figure 3, Table 3, and Figure 4 should be mentioned in respective captions. Labels should be added to the color bars shown in the Figures.

Reply: We thank the reviewer for this comment. The multiple comparison correction thresholds and color bar labels have been added to Table 2, Figure 3, Table 3, and Figure 4.

References

Catani, M., Dell'acqua, F., Vergani, F., Malik, F., Hodge, H., Roy, P., Valabregue, R., Thiebaut de Schotten, M., 2012. Short frontal lobe connections of the human brain. Cortex 48, 273-291. Dick, A.S., Tremblay, P., 2012. Beyond the arcuate fasciculus: consensus and controversy in the connectional anatomy of language. Brain 135, 3529-3550. Froudist-Walsh, S., Karolis, V., Caldinelli, C., Brittain, P.J., Kroll, J., Rodriguez-Toscano, E., Tesse, M., Colquhoun, M., Howes, O., Dell'Acqua, F., de Schotten, M.T., Murray, R.M., Williams, S.C.R., Nosarti, C., 2015. Very early brain damage leads to remodeling of the working memory system in adulthood: A combined fMRI/tractography study. Journal of Neuroscience 35, 15787-15799. Karolis, V.R., Froudist-Walsh, S., Brittain, P.J., Kroll, J., Ball, G., Edwards, A.D., Dell'Acqua, F., Williams, S.C., Murray, R.M., Nosarti, C., 2016. Reinforcement of the brain's rich-club architecture following early neurodevelopmental disruption caused by very preterm birth. Cereb Cortex 26, 1322-1335. Phillips, J.S., Greenberg, A.S., Pyles, J.A., Pathak, S.K., Behrmann, M., Schneider, W., Tarr, M.J., 2012. Coanalysis of brain structure and function using fMRI and diffusion-weighted imaging. J Vis Exp. Tseng, C.J., Froudist-Walsh, S., Brittain, P.J., Karolis, V., Caldinelli, C., Kroll, J., Counsell, S.J., Williams, S.C., Murray, R.M., Nosarti, C., 2017. A multimodal imaging study of recognition memory in very preterm born adults. Human Brain Mapping 38, 644-655. Wilkins, B., Lee, N., Gajawelli, N., Law, M., Lepore, N., 2015. Fiber estimation and tractography in diffusion MRI: Development of simulated brain images and comparison of multi-fiber analysis methods at clinical b-values. Neuroimage 109, 341-356.
